# A reliable diabetic retinopathy grading via transfer learning and ensemble learning with quadratic weighted kappa metric

**DOI:** 10.1186/s12911-024-02446-x

**Published:** 2024-02-06

**Authors:** Sai Venkatesh Chilukoti, Liqun Shan, Vijay Srinivas Tida, Anthony S. Maida, Xiali Hei

**Affiliations:** 1 School of Computing and Informatics, University of Louisiana at Lafayette, Lafayette, 70503 LA USA; 2 Department of Computer Science, College of Saint Benedict and Saint John’s University, St. Joseph, MN 56374 USA

**Keywords:** Ensemble learning, Diabetic retinopathy grading, Transfer learning, Quadratic weighted kappa, Efficient net

## Abstract

The most common eye infection in people with diabetes is diabetic retinopathy (DR). It might cause blurred vision or even total blindness. Therefore, it is essential to promote early detection to prevent or alleviate the impact of DR. However, due to the possibility that symptoms may not be noticeable in the early stages of DR, it is difficult for doctors to identify them. Therefore, numerous predictive models based on machine learning (ML) and deep learning (DL) have been developed to determine all stages of DR. However, existing DR classification models cannot classify every DR stage or use a computationally heavy approach. Common metrics such as accuracy, F1 score, precision, recall, and AUC-ROC score are not reliable for assessing DR grading. This is because they do not account for two key factors: the severity of the discrepancy between the assigned and predicted grades and the ordered nature of the DR grading scale.

This research proposes computationally efficient ensemble methods for the classification of DR. These methods leverage pre-trained model weights, reducing training time and resource requirements. In addition, data augmentation techniques are used to address data limitations, improve features, and improve generalization. This combination offers a promising approach for accurate and robust DR grading. In particular, we take advantage of transfer learning using models trained on DR data and employ CLAHE for image enhancement and Gaussian blur for noise reduction. We propose a three-layer classifier that incorporates dropout and ReLU activation. This design aims to minimize overfitting while effectively extracting features and assigning DR grades. We prioritize the Quadratic Weighted Kappa (QWK) metric due to its sensitivity to label discrepancies, which is crucial for an accurate diagnosis of DR. This combined approach achieves state-of-the-art QWK scores (0.901, 0.967 and 0.944) in the Eyepacs, Aptos, and Messidor datasets.

## Introduction

The global public health landscape is increasingly suffering from the growing prevalence of diabetes and its associated complications, leading to significant morbidity, mortality, and substantial financial expenses. Therefore, it is crucial to develop, implement, and evaluate DR prevention and treatment initiatives [1, 2]. Due to excessive blood sugar, tiny blood vessels in the retina can break and cause retinal bleeding, causing diabetic retinopathy. Any type of diabetes can result in diabetic retinopathy. The longer one has diabetes, the higher the risk of diabetic retinopathy. Depending on the severity of the disease, the effect can range from near-normal vision to complete loss of sight [3–5]. Early detection of diabetic retinopathy can prevent 95% damage to the eye. Diabetic retinopathy may not show symptoms in the early stages, as it occurs within the eye. Therefore, DR can be attacked even when blood sugar and vision are normal. Due to this, the doctor can only detect diabetic retinopathy after a proper examination [6, 7]. To prevent blindness and vision impairment, the screening for diabetic retinopathy is recommended in the WHO Global Report on Diabetes and the World Report on Vision [8].

Diabetic Retinopathy can be divided into two stages: non-proliferative diabetic retinopathy (NPDR) and proliferative diabetic retinopathy (PDR). NPDR can be divided into three phases: mild, moderate, and severe DR stages. NPDR is due to excessive sugar levels that start to affect tiny blood vessels in the retina, causing the blood vessels to become swollen and leak fluid; as a result, the retina lacks oxygen and nutrients [4, 9]. The body produces vascular endothelial growth factor (VEGF) to provide nutrients and oxygen to the retina of the eye. However, these new cells are fragile and can easily be damaged, resulting in more swelling and leakage. This advanced stage is called proliferative diabetic retinopathy (PDR), which is dangerous as it often causes complete vision loss [4, 10].

There are four main issues in the current literature on DR. One is that widely used metrics such as accuracy, F1-score, precision, and recall are inappropriate because they can be higher even when not all stages of DR are detected. Furthermore, all of these metrics do not consider the severity of disagreement between the actual and predicted labels. Second, pre-trained models such as VGG [11], ResNet [12], Inception [13], squeeze and excitation [14], AlexNet [15], and DenseNet [16], etc., are not scaled correctly in all three dimensions. Therefore, they cannot extract complex features from digital fundus imagery. The third one is that they use datasets that have an incomplete number of DR classes. The last one is that they use computationally heavy ensemble models to detect DR grade. Since treatment depends on the severity of DR, we have focused on detecting five stages of diabetic retinopathy with the highest probability possible using the EfficientNet pre-trained model. We also used pre-trained models like ResNet and VGG to showcase their inability to detect all the DR stages. Our main contributions to this paper can be summarized as follows:

1. We proposed a novel ensemble strategy that takes advantage of the model weights saved during model training. This approach is computationally efficient.
2. We investigated the effectiveness of the transfer learning method using models trained on DR datasets.
3. We perform data augmentation techniques such as CLAHE (Contrast Limited Adaptive Histogram Equalization) to improve the input images and Gaussian Blur to reduce the noise in an image.
4. We developed the classifier using the three layers, including the dropout and Relu activation layers, to reduce overfitting and improve model performance. After the first two layers extract the features of the given image, the final layer of the classifier is used to categorize the DR grades.
5. We use Quadratic Weighted Kappa (QWK) [17] as the primary DR classification metric because it considers the difference between the actual and target labels, which is crucial for DR classification.
6. By combining all the above methods, the EfficientNet-B3 model achieves a state-of-the-art QWk of 0.901, 0.967, and 0.944 on Eyepacs, Aptos, and Messidor, respectively.

The remainder of the paper can be summarized as follows: Related work section presents problems in related work. Background section describes transfer learning, QWK, and other metrics, and CLAHE, an image enhancement technique. Methodology section explains the proposed method. Results and discussion section demonstrates the details of the data set, the metric scores and the confusion matrices of the models and compares the proposed approach with the existing literature. Directions for future work section provides future directions to continue or improve the work. Conclusion section concludes the article with a summary.

## Related work

Al-Smadi et al. [18] have used the APTOS 2019 blindness detection data set [19] to classify the severity of diabetic retinopathy. They used transfer learning from six state-of-the-art models, namely ResNet-50, Inception-ResNet-V2, EfficientNet-B4, Xception, DenseNet-169, and Inception-V3. Although this method achieves strong results, its reliance on computationally demanding conventional convolution in its ensemble components limits efficiency. Furthermore, there is potential for QWK enhancement. Our method is computationally less intensive, as it uses predictions from a single model, and we obtain a higher QWK score. In the field of retinopathy research, convolutional neural networks (CNNs) have been widely used for various tasks. However, the recent emergence of Vision Transformers (ViTs) has led to an overemphasis on model complexity and scalability, often at the expense of practicality and efficiency. To address this problem, Zhu * et al.* propose a new CNN architecture, called nnMobileNet [20], specifically designed for the investigation of DR. nnMobileNet is a modified version of MobileNet that incorporates several enhancements to improve its performance and efficiency. These enhancements include channel attention, cross-layer connections, and group normalization. Channel attention allows nnMobileNet to focus on the most informative channels in the input image, while cross-layer connections facilitate better information flow between different layers of the network. Group normalization, on the other hand, enhances the stability of the network and reduces overfitting. To evaluate the effectiveness of nnMobileNet, the authors conducted extensive experiments on four public datasets. nnMobileNet can be improved using our strategy. We leave this to our future work.

Huang et al. proposes an SSiT, a novel self-supervised learning framework that uses saliency maps to effectively grade diabetic retinopathy. SSiT uses contrastive learning to enhance image representations and incorporates saliency maps to guide the learning process. Saliency-guided image classification refines the learned representations for DR grading. SSiT may require more computational resources compared to simpler supervised learning methods. Its generalizability to various data sets for the classification of DR and clinical settings remains unclear. In contrast, our proposed method is computationally efficient and has shown effectiveness in three different datasets. Matten et al. [21] provided an in-depth examination of retinal datasets, DR detection techniques, and performance evaluation metrics to identify DR. Matten et al. [22] performed an exudate detection for DR using pre-trained convolutional neural networks. The disadvantage of this paper is that the classification consists of only two classes: the presence or absence of exudate and the use of an improper evaluation metric. Matten et al. [23] proposed a method that combines the Gaussian mixture model (GMM), visual geometry group network (VGGNet), singular value decomposition (SVD), principal component analysis (PCA), and softmax for region segmentation, high-dimensional feature extraction, feature selection, and fundus image classification. Thirty-five thousand one hundred twenty-six images from the standard Eyepacs dataset were used in the experiments. The suggested VGG-19 DNN-based DR model beat AlexNet and the spatial invariant feature transform (SIFT) with respect to classification accuracy and computation time. The drawback of the paper is the use of improper evaluation metrics.

Existing DR classifiers need improvement to achieve a higher QWK. Most of the previous work focused on binary classification and did not use QWK. A QWK of 0.82 was achieved with the ensemble learning of three computationally intensive models on Eyepacs, encouraging research on resource-efficient ensemble models [18]. While Mohan et al’s [24] bi-stage feature selection model shows promise for automatic DR detection with high accuracy, it faces some limitations. Its reliance on three deep learning models for feature extraction incurs significant computational costs, potentially hindering its adoption in resource-constrained settings. Furthermore, the model’s performance was not evaluated using the QWK metric, which is crucial for imbalanced datasets like DR. This lack of evaluation leaves a gap in understanding how the model would perform under real-world conditions. Despite these shortcomings, the model’s strong results on public datasets suggest its potential. With further development and optimization to address computational efficiency and ensure robust performance in practical scenarios.

While Mohan et al’s [25] federated learning approach (DRFL) promises high accuracy for automated DR detection while protecting patient data privacy. But it faces some limitations, such as the fact that combining data from diverse institutions can introduce heterogeneity, potentially making the model less generalizable to different populations. Additionally, the central server used for feature extraction creates a vulnerability and represents a computational bottleneck. Further evaluation on more diverse real-world datasets and with imbalance-sensitive metrics like QWK is crucial to understanding DRFL’s performance under realistic conditions. Privacy concerns about information leakage through gradients also require further investigation.

QWK is a metric that measures the agreement between two raters in multiclass classification problems with ordinal labels. It is robust to unbalanced data. EfficientNet is a family of convolutional neural networks (CNNs) designed to be accurate and efficient. They are based on the principle of compound scaling, which means that all dimensions of the network (depth, width, and resolution) are scaled together in a coordinated way. This ensures that the network remains balanced and efficient. In this study, we used QWK as the primary evaluation metric because the grades of diabetic retinopathy (DR) are ordinal, which means they can be classified from mild to severe. We show in Table 5 and Metric scores on Reduced Eyepacs dataset section that EfficientNet has an advantage over models such as VGG and ResNet, which are scaled according to their depth. EfficientNet can detect multiple classes, which is an advantage. As shown in the literature review, ensemble transfer learning techniques are more effective in DR grading. Therefore, in our study, we used QWK, transfer learning, EfficientNet, and resource-efficient ensemble techniques.

## Background

### Transfer learning

In deep learning, we can speed up learning by transferring knowledge from related tasks. This involves choosing a pre-trained model, one that has proven itself on large datasets such as ImageNet [26]. We then tweak the final classification layer to focus on the specific task at hand. Several pre-trained models are available, which were trained on ImageNet [26]. We have taken VGG [11], ResNet [12], and EfficientNet [27] because they have been widely used in academic research and industry. Their extensive usage is because the skip connections introduced in ResNet enhance the trainability of deeper networks. The lower-dimensional filters used in the VGG network resulted in fewer trainable parameters in the filter. Furthermore, an efficient compound scaling method in EfficientNet effectively scales the height, width, and depth of the network to learn the most complex features.

Simonian et al. [11] have proposed a CNN architecture known as Visual Geometry Group (VGG). In this paper, they have used only 3$\times$3 convolutional filters. This architecture has secured the first and second places in the localization and classification tasks in the ImageNet Challenge 2014. In the initial layers, they applied the 3$\times$3 filter twice consecutively, which has a reception field similar to using the 5$\times$5 filter once. In the final layers, they applied the $3\times$3 filter three times sequentially, which has a reception field similar to that obtained by applying the 7$\times$7 filter once. There are two main advantages to stacking small filters such as $3\times 3$ instead of larger ones. One is that the number of trainable parameters gets reduced, and the second is that more nonlinear activation functions can be used between the convolution operations, increasing the model’s learning power.

He et al. [12] have proposed deep residual learning (ResNet) for image recognition. Before ResNet was introduced, the deeper models were harder to optimize, which led to a poorer performance compared to the shallower models. Therefore, to overcome this, ResNet introduced skip connections, which made it easier to train deeper models and had better performance than shallower models. Because the models can be developed more deeply, they have achieved better accuracy in the ImageNet data set [26]. In addition, they have developed layers with a depth of up to 152 layers. As a result, ResNet came in first place in the ILSVRC 2015 classification task.

Tan et al. [27] have developed EfficientNet. EfficientNet is developed using the compound scaling method that uniformly scales depth, width, and resolution dimensions using a simple and effective compound coefficient. The authors proposed a baseline CNN using Neural Architecture Search (NAS) and then scaled it up to obtain a family of models known as EfficientNets, which have achieved better accuracy and efficiency than previous convolutional networks. The baseline network is composed of depth-wise separable convolutions, which helps reduce the number of trainable parameters in a model. Squeeze and excitation are used to learn the interdependencies of the channels. Specifically, EfficientNet-B7 achieves 84.4% top-1 and 97.1% top-5 accuracy on ImageNet while being 6.1 times faster and 8.4 times smaller on inference than the best CNN in use before EfficientNet.

### Quadratic Weighted Kappa (QWK)

QWK highlights the level of disagreement between actual and predicted labels. Using three matrices, namely the expected matrix (E), the output matrix (O) and the weighted matrix (W), the quadratic weighted kappa can be computed as follows:

**Step 1:** Calculate the expected matrix (E) taking the outer product between the actual (A) and predicted (P) label vectors.

1 $$\begin{aligned} E = A \otimes P \end{aligned}$$

**Step 2:** Construct the output matrix by building a confusion matrix of actual and predicted labels.

**Step 3:** Calculate the weight matrix as follows:

2 $$\begin{aligned} {W_{ij}} = {\frac{(i -j)^2}{(k -1)^2}} \end{aligned}$$

where *i* is an actual label, *j* is the predicted label and *k* is the number of classes.

**Step 4:** Normalize the expectation matrix (E) and output matrix (O) as follows:

3 $$\begin{aligned} E = \frac{E}{\Sigma _{ij}E_{ij}} \end{aligned}$$

4 $$\begin{aligned} O = \frac{O}{\Sigma _{ij}O_{ij}} \end{aligned}$$

**Step 5:** Calculate the weighted kappa using the following formula:

5 $$\begin{aligned} Quadratic \ \ Weighted \ \ Kappa = {1 - \frac{num}{den}} \end{aligned}$$

Where *num* is the sum of elements obtained using element-wise multiplication between weight matrix (W) and output matrix (O), and *den* is the sum of elements obtained using element-wise multiplication between weight matrix (W) and expectation matrix (E).

Quadratic weighted kappa can range from -1 to 1. The larger the quadratic weighted kappa, the lower the disagreement between a predicted and an actual label. The metric score of 1 is a perfect level of agreement, whereas the metric score of -1 is an extreme disagreement. QWK assumes that the target and predicted labels are ordinal. The discrepancy depends on the variation between the target and predicted labels. For example, if the predicted label is 0 and the actual label is 2, then the level of disagreement is double that of when the actual label is two and the predicted label is 1. QWK is particularly well suited to assess tasks where labels are in natural order, such as grading the severity of Diabetic Retinopathy (DR). It not only measures the agreement between predicted and actual labels but also accounts for the severity of disagreements, assigning higher penalties to larger discrepancies. This makes it more sensitive to errors that could have significant clinical implications in the diagnosis of DR. An accurate classification of the severity of DR is essential for the appropriate treatment and management of the condition. Misclassifications, especially those that underestimate the severity, could lead to delayed interventions and potential vision loss. The ability of QWK to capture both the direction and magnitude of disagreements makes it a more reliable metric for assessing performance in this context.

### Contrast Limited Adaptive Histogram Equalization (CLAHE)

The classification of diabetic retinopathy (DR) is highly dependent on the visibility of subtle features in fundus images. However, uneven illumination and inherent low contrast often obscure these critical details, leading to misdiagnosis and delayed treatment. CLAHE focuses on local image regions, improving contrast and revealing hidden microaneurysms, hemorrhages, and exudates crucial for an accurate classification of DR. The adaptive nature of CLAHE preserves natural image details, while employing a clever "clip limit" to suppress noise amplification, ensuring clear and information-rich details. Local equalization of CLAHE enhances the contrast between the retinal lesions and the surrounding background. This makes subtle lesions, such as drusen or microaneurysms, more prominent and easier to detect. CLAHE can address non-uniform illumination issues common in fundus images, further improving lesion visibility. This is especially helpful for images acquired under different lighting conditions or with uneven camera response. CLAHE's histogram manipulation can partially suppress noise in fundus images, leading to cleaner visualizations.

## Methodology

### The proposed method

This study uses transfer learning based on VGG, ResNet, and EfficientNet to implement the diabetes retinopathy classification task. We developed the classifier to classify the DR grades. We trained both the feature extractor and the classifier during the training phase. We propose the two ensemble strategies using a single model. Furthermore, we investigate the impact of using transfer learning from models trained on DR grading. We provide the details of the EfficientNet architecture in the next section, as it achieved the best performance among the different pre-trained models used in this study.

#### EffcicientNet architecture

**Fig. 1:**
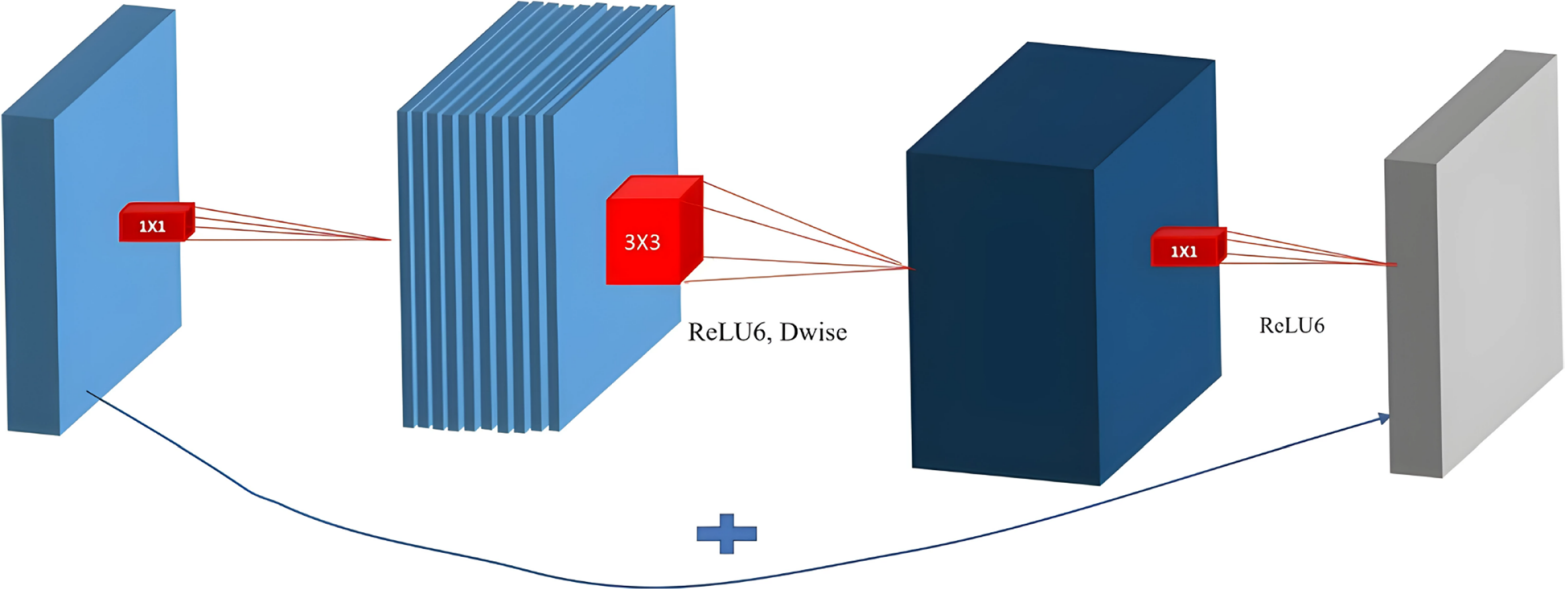
Inverted Residual block

This section describes the details of the EfficientNet architecture. Table 1 illustrates the details of the architecture of the baseline model of the EfficientNet family, which is EfficientNet-B0. The main building block is the inverted residual block [28], as illustrated in Fig. 1, which is indicated as MBCONV in Table 1. The squeeze and excitation technique [14] is used to build MBCONV. In squeeze and excitation, the input feature maps are increased depth-wise using $1\times 1$ convolutions. Then, $3\times 3$ depth-wise and point-wise convolutions are performed to reduce the channels in the output feature map. Finally, the short connection connects the input and output feature maps. The main motivation for the authors of EfficientNet [27] is to improve classification efficiency and accuracy. To obtain this, it is essential to balance the depth, width, and resolution of the network while scaling. Therefore, an effective compound scaling method is developed and can be formulated as in Eq. 6.

6 $$\begin{aligned} &depth : d = \alpha {^\phi }\\&width : w = \beta {^\phi }\\&resolution : r = \gamma {^\phi }\\&s.t. \alpha . \beta ^2 . \gamma ^2 \approx 2\\&\alpha \ge 1,\beta \ge 1,\gamma \ge 1 \end{aligned}$$

**Table 1:** EffcientNet baseline model architecture

$stage_{i}$	$F_{i}$	$H_{i} \times W_{i}$	*C* *i*	$L_{i}$
1	Conv 3$\times$3	224$\times$224	32	1
2	MBConv1, k3$\times$3	112$\times$112	16	1
3	MBConv6, k3$\times$3	112$\times$112	24	2
4	MBConv6, k5$\times$5	56$\times$56	40	2
5	MBConv6, k3$\times$3	28$\times$28	80	3
6	MBConv6, k5$\times$5	14$\times$14	112	3
7	MBConv6, k5$\times$5	14$\times$14	192	4
8	MBConv6, k3$\times$3	7$\times$7	320	1
9	Conv 1$\times$1 & Pooling & FC	7$\times$7	1,280	1

By setting $\phi$ equal to 1 and using the grid search method, the parameters $\alpha$, $\beta$ and $\gamma$ can be found by choosing the parameters that give the best accuracy. Then, by increasing $\phi$, the higher versions of the EfficientNet models were developed.

#### Final classifier modeling

**Fig. 2:**
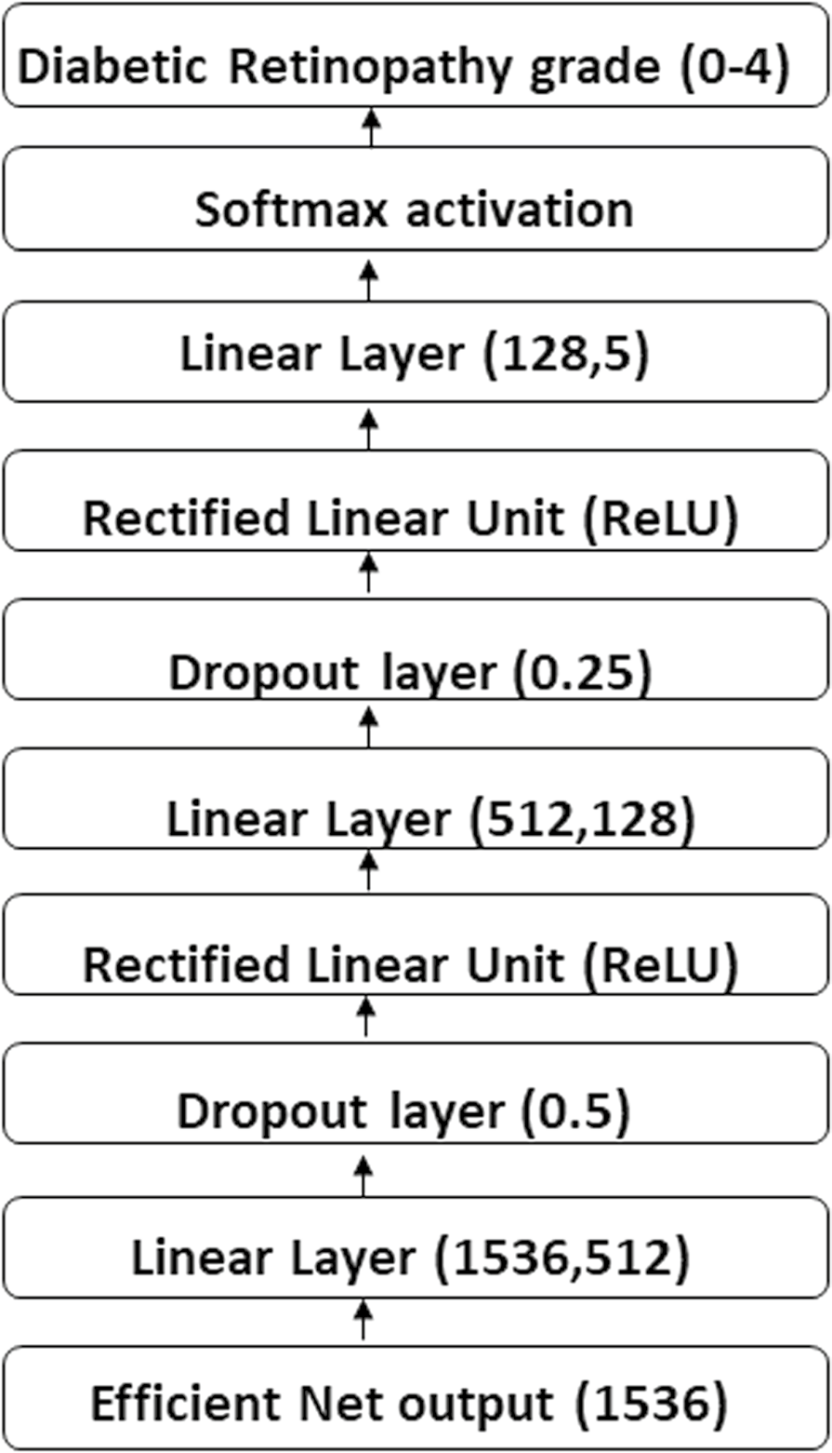
The Architecture of the Final Model

Figure 2 represents the final classifier we developed for DR classification. We use EfficientNet, VGG, and ResNet models. Of all the models, EfficientNet-B3 achieved a state-of-the-art result. The classifier is developed as follows: First, EfficientNet-B3 produces a size feature: $7 \times 7 \times 1,536$. Then it uses the adaptive average pooling 2d of output size $1\times 1$ to generate the feature of size $1\times 1\times$1,536; it is then flattened to have a size of 1,536, which will be fed to the classifier. We replace the classifier used in EfficientNet-B3 with the final classifier developed to detect five stages of diabetic retinopathy. First, the feature vector of dimension 1,536 has been fed to the fully connected layer, giving a feature vector of size 512. Next, it is passed to the dropout layer, which has a drop rate of 0.5, and the ReLU activation layer. Next, the ReLU output is passed to the fully connected layer, giving the output size of 512, which is fed to the dropout layer with a drop rate equal to 0.25 and then to the ReLU activation layer. Finally, the output of the ReLU activation layer is given to the fully connected layer, which has five units equal to the number of output classes. Then it is fed to the Softmax activation layer to generate the probabilities for each category. The same classifier structure is used to train all the pre-trained models considered in this article.

#### Hyper-parameter tuning

To improve model performance, we used Adam optimizer, which combines the capabilities of both RMSProp and momentum. Furthermore, to avoid exploding gradients, we used gradient clipping with clipping equal to 0.1 and a weight decay of 10^-4^. The learning rate for all models used in this article is 0.001.

#### First ensemble strategy

**Fig. 3:**
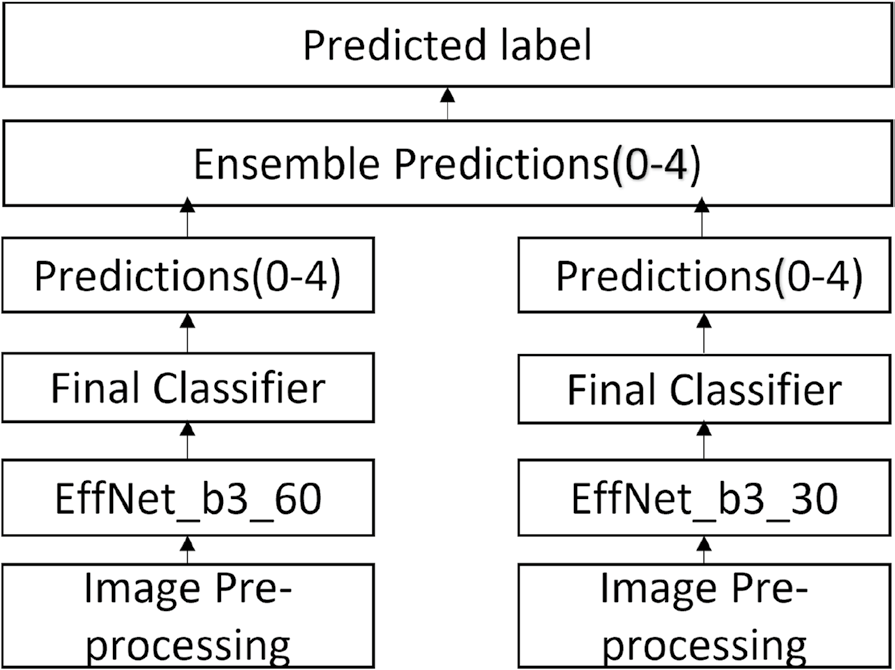
Illustration of first ensemble strategy

Figure 3 illustrates the first ensemble strategy. The EfficientNet-B3 model is trained for 60 epochs. Weights are saved at 30 and 60 epochs, and then the two models are created. The predictions are then obtained from the models. Finally, we aggregated the two sets of predictions to obtain the final predictions, which were used to compute the evaluation metrics used in the paper. In the strategy, only normalization and resizing are implemented. All images were resized to $150 \times 150$.

#### Second ensemble strategy

For the second ensemble strategy, we tested different data augmentations and found that CLAHE (Contrast limited Adaptive Histogram Equalization) in combination with Gaussian Blur performs well. We do normalization and the images are resized to $300 \times 300$.

In this second ensemble strategy, the model weights are saved at 10 intervals during training. At the time of inference, the ten predictions are obtained using the saved model weights at ten intervals. The final prediction is estimated to be the most common prediction among all the predictions.

#### Impact of transfer learning using models trained on DR dataset

In this project, we use three different datasets, namely, a resized version of the Eyepacs dataset, the Aptos 2019 data set, and the Messidor2. The Eyepacs dataset is the largest. Therefore, we used the model trained on the Eyepacs data set to fine-tune on the Aptos and Messidor databases to see if this helped. We provide the results in the next section.

## Results and discussion

We use the PyTorch framework that supports automatic differentiation and efficiently uses GPUs for parallel processing [29]. As a result, PyTorch makes deep learning model training simpler and faster. Furthermore, we used Google Colab Pro, which gives GPU run time for a limited time and RAM of 25.46 GB, to train the developed models.

Next, we describe the details of the data set and the metric scores, such as accuracy, precision, recall, F1-score, confusion matrices, and QWK.

### Dataset description

This project uses the three datasets. First, a resized version of the Eyepacs dataset for diabetic retinopathy [30] comprises 35,126 images of the retinal digital fundus and high-resolution digital fundus images of the retina taken under different imaging conditions. We divide the data into three datasets for training, validation, and testing. The training set contains 24,590 retinal images, while the validation and test data sets contain 5,268 retinal images each. Table 2 describes the details of the data set that we used in this work. It has five target labels: the right and left eyes of each class and the number of images for each category; output labels indicated as 0-4, label counts describe the number of images for each class; and the target class denotes the grade of diabetic retinopathy. The grade can help doctors diagnose the patient with the appropriate treatment, as the treatment depends on the severity of diabetic retinopathy. The last two columns correspond to the left and right of an image for each stage of diabetic retinopathy.

**Table 2:** Dataset Description

Output labels	Label counts	Target Class	Left Eye	Right Eye
0	25,180	No DR		
1	2,443	Mild DR		
2	5,292	Moderate DR		
3	873	severe DR		
4	708	Proliferate DR		

The second is the Asia Pacific Tele-Ophthalmology Society 2019 blindness detection data set (APTOS 2019 BD), which is a collection of 3,662 fundus photographs of patients from rural India. The data set was created by Aravind Eye Hospital in India and contains images collected under varying conditions and environments over a long period. The images were labeled by a group of trained physicians using the International Clinical Diabetic Retinopathy Disease Severity Scale (ICDRSS). The data set includes five categories of DR: non-DR, mild DR, moderate DR, severe DR, and proliferative DR. The last one is that the Messidor-2 data set comprises 874 DR examinations, totaling 1,748 fundus images. These images were acquired from 762 patients with a wide range of levels of severity of DR, including No DR, mild DR, moderate DR, severe DR, and proliferative DR.

### Metric scores on Reduced Eyepacs dataset

Table 3 shows the accuracy, F1-score, precision, recall, AUC-ROC score, and QWK of the VGG, ResNet, and EfficientNet models. The EfficientNet-B3 model trained for 30 and 60 epochs is denoted as EffNet-B3_30 and EffNet-B3_60, respectively. The efficient net model that is obtained using our first ensemble strategy is represented as EffNet-B3_En. The quadratic-weighted kappa is defined as QWK.

**Table 3:** Metrics scores versus the model

Model	Accu.	F1-score	Prec.	QWK	Recall	AUC-ROC score
VGG-16	0.732	0.625	0.533	0	0.726	0.196
VGG-19	0.734	0.617	0.544	0	0.732	0.196
ResNet-18	0.723	0.614	0.525	0	0.714	0.196
ResNet-34	0.724	0.612	0.512	0	0.723	0.196
ResNet-50	0.735	0.623	0.534	0	0.726	0.196
ResNet-101	0.766	0.613	0.556	0.009	0.73	0.196
ResNet-152	0.721	0.612	0.578	0.067	0.723	0.196
EffNet-B0	0.767	0.721	0.689	0.501	0.767	0.332
EffNet-B1	0.778	0.722	0.689	0.478	0.778	0.312
EffNet-B2	0.767	0.712	0.667	0.502	0.767	0.267
EffNet-B3_30	0.852	0.844	0.833	0.824	0.845	0.478
EffNet-B3_60	0.867	0.842	0.854	0.854	0.867	0.601
EffNet-B3_En	**0.924**	**0.867**	**0.867**	**0.867**	**0.878**	**0.742**
EffNet-B4	0.767	0.701	0.694	0.401	0.767	0.332
EffNet-B5	0.721	0.612	0.521	0	0.722	0.343
EffNet-B6	0.767	0.723	0.701	0.532	0.767	0.312

The Table 3 shows that the QWK of VGG-16, VGG-19, ResNet-18, ResNet-34, and ResNet-50 is absolute zero due to the strong disagreement between the actual and predicted labels. The QWK score of 0 is because the given images were taken under various imaging conditions and the models are not robust to varying imaging conditions. Furthermore, the different versions of the ResNet and VGG models are only scaled by depth, which is not enough to extract complex features in the input images. However, the higher versions of ResNet, which are ResNet101 and ResNet152, give a slightly positive QWK score, indicating that increasing the depth will increase the agreement between the actual labels, which is desirable. Observing the QWK scores for the VGG and ResNet models, we found that the models scaled only by depth are ineffective in extracting complex image features. Thus, we selected the EfficientNet models that use the compound scaling method to scale depth, width, and image resolution.

From Table 3, it is visible that the QWK score for all versions of EfficientNet is comparatively good. Of all the versions, the EfficientNet-B3 ensemble has performed very well, as indicated by the highest QWK score. The reason is that different versions of EfficientNet have different resolutions, depths, and widths. The EfficientNet-B3 model’s resolution, depth, and width scaling parameters are suited to the given input-resized images. However, we observed that even if the resolution, depth and width are scaled more than needed, they will underperform, which is evident from the QWK scores of the EfficientNet-B4, EfficientNet-B5 and EfficientNet-B6 models. The reason for picking the QWK is that even when the model has high accuracy, recall, etc., it is not reliable unless it has a high QWK metric score. Even if the model cannot detect all stages, the accuracy, precision, recall, and F1-score will be much higher. In contrast, the QWK score is much lower.

**Fig. 4:**
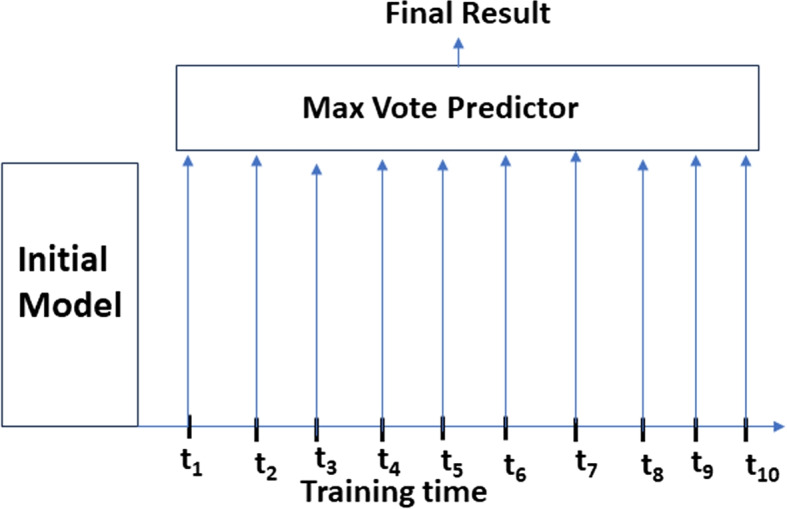
Illustration of second ensemble strategy

In summary, the QWK scores of the lower-depth VGG and ResNet models are exactly 0. Resenet models with greater depth show positive QWK. However, due to compound scaling, the most EfficientNet models received higher QWK scores, highlighting the significance of choosing the appropriate pre-trained model. The QWK score of 0.867 is obtained using the predictions of the EfficientNet model based on the first ensemble strategy. Similarly, we can see that the ensemble EfficientNet model has higher accuracy, F1-score, precision, recall, and AUC-ROC score. The reason for this is that the EfficientNet-B3_en model used the knowledge from two different model weights. The AUC-ROC score for all VGG and ResNet models is 0.2 because these models can only detect one DR stage. This is clearly shown in Fig. 4, which shows the confusion matrices for the different models. Moreover, the AUC-ROC can be sensitive to class imbalance. This is because the AUC-ROC curve is calculated by averaging the TPRs for each class. If one class is much more common than the others, then the TPR for that class will have a great impact on the overall AUC-ROC score.

The explanation for low QWK scores and zero QWK scores is given by using confusion matrices and definitions of TP, FP, FN, and TN for the five-class DR grading. An explanation is provided for why efficient models, especially EfficientNet-B3 models, received higher QWK ratings. Table 4 represents the confusion matrix of the ensemble EfficientNet-B3 model. The following steps illustrate how to calculate True Positive (TP), False Positive (FP), True Negative (TN), and False Negative (FN) for the No DR class of the ensemble Efficientnet-B3 model.

**Table 4:** Confusion Matrix for ensembled EfficientNet-B3

Predicted Result		0	1	2	3	4
Real Result	0	**3886**	1	26	0	0
	1	169	**80**	91	1	0
	2	68	6	**710**	1	7
	3	2	0	31	**93**	5
	4	3	0	7	3	**98**

**TP:** The actual and predicted labels should be the same. So, for the No DR class, the TP is 3,886.

**FN:** The sum of all cells in the corresponding row except the first cell, which is 1 + 26 + 0 + 0 = 27.

**FP:** The sum of all cells in the corresponding column except the first cell is 169 + 68 + 2 + 3 = 242.

**TN:** The sum of all the remaining cells is 80 + 91 + 1 + 0 + 6 + 710 + 1 + 7 + 0 + 31 + 93 + 5 + 0 + 7 + 3 + 98 = 2784.

TP, TN, FP, and FN can be calculated in the same way for the remaining classes.

Table 5 demonstrates the confusion matrices for all the models used in this project in addition to the ensemble Efficient-B3 model. We found that the QWK score of the VGG and ResNet models is either 0 or very low because the models only identify class 0 and completely misclassify the remaining classes. Unlike them, the EfficientNet models could also recognize the other classes. Moreover, the EfficientNet-B3_30, EfficientNet-B3_60, and ensemble EfficientNet-B3 model have detected all classes with high probability. Hence, the QWK score is very high for the EfficientNet-B3 models compared to the VGG, ResNet, and other EfficientNet models.

**Table 5:** Confusion matrices of VGG, ResNet, and Efficient model

Model Name	VGG-16						VGG-19					ResNet-18					ResNet-34					ResNet-50				
Real Result	Predicted Result																									
		0	1	2	3	4	0	1	2	3	4	0	1	2	3	4	0	1	2	3	4	0	1	2	3	4
	0	**3638**	0	0	0	0	**3887**	0	0	0	0	**3825**	0	0	0	0	**3830**	0	0	0	0	**3864**	0	0	0	0
	1	363	**0**	0	0	0	376	**0**	0	0	0	371	**0**	0	0	0	391	**0**	0	0	0	365	**0**	0	0	0
	2	763	0	**0**	0	0	793	0	**0**	0	0	806	0	**0**	0	0	804	0	**0**	0	0	806	0	**0**	0	0
	3	131	0	0	**0**	0	131	0	0	**0**	0	152	0	0	**0**	0	138	0	0	**0**	0	116	0	0	**0**	0
	4	113	0	0	0	**0**	113	0	0	0	**0**	114	0	0	0	**0**	105	0	0	0	**0**	117	0	0	0	**0**
Model Name	ResNet-101						ResNet-152					EfficientNet-B0					EfficientNet-B1					EfficientNet-B2				
Real Result	Predicted Result																									
		0	1	2	3	4	0	1	2	3	4	0	1	2	3	4	0	1	2	3	4	0	1	2	3	4
	0	**3848**	0	0	0	0	**3775**	0	0	0	0	**3787**	0	105	2	5	**3833**	0	68	0	0	**3761**	0	120	0	0
	1	383	**0**	0	0	0	360	**0**	0	0	0	321	**0**	20	0	0	357	**0**	17	0	0	346	**0**	29	0	0
	2	783	0	**0**	0	0	814	0	**0**	0	0	532	0	**228**	25	1	501	0	**273**	0	2	487	0	307	0	0
	3	140	0	0	**0**	0	128	0	0	**0**	0	27	0	67	**39**	2	30	0	89	**0**	3	24	0	86	**0**	0
	4	103	0	0	0	**0**	90	0	0	0	**0**	28	0	47	17	**15**	34	0	41	0	**18**	17	0	91	0	**0**
Model Name	EfficientNet-B3_30						EfficientNet-B3_60					EfficientNet-B4					EfficientNet-B5					EfficientNet-B6				
Real Result	Predicted Result																									
		0	1	2	3	4	0	1	2	3	4	0	1	2	3	4	0	1	2	3	4	0	1	2	3	4
	0	**3868**	3	20	0	0	**3772**	8	65	0	0	**3851**	0	38	0	2	**3726**	0	83	1	0	**3809**	0	59	0	2
	1	326	**23**	34	0	0	244	**31**	128	0	0	348	**0**	3	0	0	363	**0**	11	0	0	335	**0**	13	0	0
	2	247	34	**501**	0	0	114	9	**624**	5	0	605	0	**194**	5	8	587	0	**234**	21	0	515	0	**269**	**1**	10
	3	10	0	**69**	38	0	3	0	77	**46**	13	42	0	71	**46**	2	36	0	70	**33**	0	26	0	87	**5**	18
	4	16	0	29	15	**37**	9	0	14	6	**98**	44	0	58	6	**16**	19	0	65	21	**0**	33	0	66	1	**21**

### Results of model trained on three datasets

In this section, we discuss and analyze the results of the model trained using our second ensemble strategy. Then, we investigate the impact of transfer learning using a model pre-trained on DR dataset. All of these models were pre-processed using CLAHE, Gaussian Blur, and normalization. For a robust evaluation of our strategy, we evaluated our methods on three datasets, such as the resized and reduced version of the EyePacs, Aptos, and Messidor-2 databases. Table 6 shows the results of the second ensemble strategy and also the impact of transfer learning (TL) on the models trained in the DR datasets. The pre-trained model in Table 6 indicates whether a model is pre-trained in the DR data set. However, the ensemble indicates whether or not our second ensemble strategy is used. It is evident from Table 6 that our second ensemble strategy performs better than directly using the pre-trained model. For example, on the Aptos dataset, when a model pre-trained on Eyepacs is used, the QWK score of 0.967 is obtained when our ensemble strategy is performed. On the contrary, it achieves 0.956 when the ensemble strategy is not used. Moreover, it is clearly shown in Table 6 that a model pre-trained on the DR dataset has better performance than directly using the publicly available model. For example, on the Messidor2 database, the publicly available model got a QWK score of only 0.854, whereas the model pre-trained on EyePacs has a QWK score of 0.933.

**Table 6:** Results on three datasets using proposed strategies

Dataset	Pretrained Model	Ensemble	Accuracy	Precision	Recall	F1-score	QWK
Eyepacs	None	No	0.921	0.923	0.923	0.924	0.878
**Eyepacs**	**None**	**Yes**	**0.944**	**0.943**	**0.942**	**0.932**	**0.901**
Aptos	None	No	0.942	0.943	0.943	0.944	0.954
Aptos	Eyepacs	No	0.954	0.952	0.942	0.954	0.956
**Aptos**	**Eyepacs**	**Yes**	**0.954**	**0.953**	**0.95**	**0.956**	**0.967**
Messidor2	None	No	0.854	0.856	0.853	0.844	0.854
Messidor2	Eyepacs	No	0.923	0.922	0.923	0.912	0.933
**Messidor2**	**Eyepacs**	**Yes**	**0.924**	**0.922**	**0.923**	**0.921**	**0.944**

### Comparisons with existing literature

This section contrasts our second ensemble strategy, the transfer learning impact, with previous work. We describe the most recent and related work to compare our approach. We present the papers that considered QWK, as it is the main evaluation metric for DR grading. Table 7 compares the results of the existing work with our work.

**Table 7:** Comparision of Results with existing literature

Paper	Author	year	model	Dataset	QWK
[18]	Al Smadi et al.	2021	Ensemble (DenseNet-169, Inception, Xception)	Eyepacs	0.824
[20]	Wenhui Zhu et al.	2023	nnMobileNet	Aptos	0.934
				Messidor-2	0.913
[31]	Yijin Huang et al.	2023	SSiT	APTOS	0.925
				Messidor-2	0.799
Ours	Chilukoti et al.	-	Proposed model	Eyepacs	0.901
				APTOS	0.967
				Messidor-2	0.944

Al Smadi et al. [18] used the Eyepacs competition data set and developed an Ensemble model using DenseNet-169, Inception-V3 and Xception [32], which obtained a QWK score of 0.824.

Wenhui Zhu et al. [20] developed the nnMobileNet model and evaluated it in the Aptos and Messidor-2 databases and obtained QWK scores of 0.925 and 0.913, respectively.

Yijin Huang et al. [31] The author developed the SSiT model, whose performance was evaluated in the Aptos and Messidor-2 databases, producing QWK scores of 0.925 and 0.799, respectively.

From Table 7 we can see that our model and strategies have state-of-the-art QWK scores of 0.901, 0.967, and 0.944 in Eyepacs, Aptos, and Messidor-2, respectively.

## Directions for future work

More advanced architectures, such as CoAtNet [33], which uses depth-wise convolution and self-attention [34], can further improve QWK. The proposed work can be further extended using Federated Learning (FL) [35] that uses a single data set for each client and obtains the best global model instead of training the model independently on multiple datasets. Furthermore, federated learning helps to protect the privacy of patients who contributed to the dataset, since only model weights are shared rather than the data. Moreover, local differential privacy can be used in FL to improve the client’s privacy. Our methods and strategies can improve the performance of nnMobilent.

## Conclusion

The research presented here proposes computationally efficient ensemble models that take advantage of the model weights saved during training for DR classification. It investigates the impact of transfer learning from pre-trained DR models, finding significant improvements in grading accuracy. To enhance image quality and reduce noise, data augmentation techniques such as CLAHE and Gaussian Blur are employed. A three-layer classifier is developed that incorporates dropout and ReLU activation to mitigate overfitting and improve generalization. The first two layers extract features from the input images, while the final layer classifies the DR grade. By prioritizing QWK, which rewards accurate predictions and penalizes large discrepancies, the models reached state-of-the-art scores of 0.901, 0.967, and 0.944 on the Eyepacs, Aptos, and Messidor datasets.

## Data Availability

The datasets used in this project are publicly available and can be found at [30, 36, 37].
